# Deficit in Decision-Making in Chronic, Stable Schizophrenia: From a Reward and Punishment Perspective

**DOI:** 10.4306/pi.2009.6.1.26

**Published:** 2009-03-31

**Authors:** Yang Tae Kim, Kyoung-Uk Lee, Seung Jae Lee

**Affiliations:** 1 Department of Psychiatry, Bugok National Hospital, Changnyeong, Korea.; 2 Department of Psychiatry, Uijeongbu St. Mary's Hosptial, The Catholic University of Korea, College of Medicine, Uijeongbu, Korea.; 3 Department of Psychiatry, School of Medicine, Kyungpook National University, Daegu, Korea.

**Keywords:** Schizophrenia, Decision-making, Gambling task, Reward, Punishment

## Abstract

**Objective:**

We compared patients with chronic schizophrenia and normal controls with respect to decision-making ability. Measures were implemented to control for the participants' intelligence levels as well as to ensure to use of a moderate sample size. The goal of this study was to confirm inconsistent results from previous studies which had stemmed from too small of a sample size, highly variable performance of normal controls, and not controlling for intelligence as a confounding factor.

**Methods:**

Fifty-two chronic stable schizophrenic inpatients and 55 healthy controls participated in the study. We controlled for intelligence by including subjects with intelligence quotient's (IQ) between 80 and 120, examining any differences in decision-making performance between groups on the Iowa Gambling Task (IGT). We also addressed several issues relating to performance on the IGT, such as working memory and clinical symptoms.

**Results:**

Schizophrenic patients were found to perform poorly on the IGT relative to normal controls (F_1,105_=17.73, p<0.001); however, more importantly, they also displayed the slow yet profitable shift from disadvantageous decks to advantageous decks over time. We also found that when compared with healthy controls, schizophrenic patients showed a poorer performance on the Wisconsin Card Sorting Test (WCST)(t=-5.48, p<0.001 for perseverative error) which was not related to their performance on the IGT.

**Conclusion:**

Based on previous literature and the results of this study, impaired sensitivity to both reward and punishment might be a more plausible explanation for the poor performance on the IGT in the schizophrenic group. We speculated that this impairment seemed related more to the different responsiveness to the magnitude than to the frequency of punishment, and to the different interpretation of less informative verbal cues in the context of the reinforcing schedule.

## Introduction

Cognitive dysfunction has been described as a hallmark feature of schizophrenia since the first descriptions of the illness.^1^ Due to recent advances in neuropsychological assessment and neuroimaging techniques, cognitive impairment has again been established as one important component of the pathophysiology of schizophrenia.

Patients with schizophrenia show deficits in a variety of cognitive domains.^2^,^3^ In general, deficits are observed on the tests of the higher cognitive functions, such as sustained attention, executive function, working memory, language skills, explicit learning and memory, and perceptual motor processing.^4^,^5^ Goldman-Rakic's work lead to the proposal that the prefrontal cortex might be the primary site of schizophrenia pathology, affecting working memory in particular and leading to avolition, behavioral disorganization, conceptual thinking, and memory formation.^6^,^7^

In addition, there is growing evidence that schizophrenic patients show emotional disturbances and social dysfunction,^8^-^10^ which could be explained, to some extent, by impaired decision-making processes. This decision-making process would be involved in interpersonal interaction and social situations.^11^ In general, decisions are made by assessing reward and punishment based on both cognitive and affective information.^12^ Some judgments are made under explicit (cognitive, conceptual) awareness, whereas others are made under ambiguous situations depending more on affective information.^12^

The Iowa Gambling Task (IGT) was developed to assess the substantial role of the affective aspect in decision-making.^13^ In this task, subjects are presented with four decks of cards and are asked to choose any deck, in any sequence, and then take a card from it. They win or lose money with each turn of a card. Participants do not appear, subjectively, to understand the contingencies of the game. Nevertheless, they can quite rapidly develop a "feeling" about which decks are good or bad. Thus, participants seem to acquire normal performance by relying on an emotionally mediated "feeling" or "hunch" in the absence of conceptual awareness.

To investigate the role of decision-making on social dysfunction in schizophrenia, there have already been several studies based on the performance of the IGT in schizophrenic patients, most of which have produced inconsistent results. Wilder et al.^14^ showed that the performance of schizophrenic patients is relatively uncompromised. Three other studies found that schizophrenia performed significantly worse on the IGT than normal controls.^15^-^17^ With respect to the relationship between executive function and the IGT, Cavallaro et al.^18^ proposed a dissociation of the Wisconsin Card Sorting Test (WCST) and IGT performances based on the findings that schizophrenic patients performed to the same level as healthy controls on the IGT but significantly worse than healthy controls on the WCST.

Despite these findings, previous studies have exhibited several limitations. Firstly, the number of subjects who participated in these studies was small (less than 20).^14^,^15^,^17^,^19^ The control groups also posed a problem, as they performed notably better in the IGT than groups of healthy volunteers in two other studies which described a pattern of impaired performance on decision-making in schizophrenic patients.^16^,^17^ That is, the statistical significance of these differences might be given by the scores of normal controls. Secondly, some studies did control for intelligence as a confounding factor.^14^,^16^,^18^,^19^ Given the reduced overall intelligence in patients with schizophrenia,^20^ the interpretation of their results may be less evident whether the given deficit is selective or is part of more generalized neuropsychological impairment. Bechara, a developer of this task, mentioned "intelligence and memory do indeed impact on decision-making, when it is in a defective range."^21^

In the current study, we controlled for intelligence as a confounding factor by including healthy subjects with IQ's between 80 and 120, then, examining the performance of decision-making on the IGT between groups with moderate size sample. This study also addressed several issues relating to performance on the IGT, such as working memory and clinical symptoms, in addition to further expanding upon the theory of reward and punishment in schizophrenia.

## Methods

### Subjects

Fifty-two stable schizophrenic inpatients and 55 healthy control subjects were recruited from Bugok National Hospital and Uijeongbu St. Mary's Hospital. The Catholic University of Korea and the Institutional Review Board approved this study. After receiving a complete description of the study, written informed consent was obtained from all participating subjects.

Fifty-two chronic stable schizophrenic inpatients who were about to discharge after remission or who were participating in open ward-based rehabilitation programs were recruited from Bugok National Hospital. Psychiatric subjects met the following inclusion criteria: 1) age 20-50, 2) diagnosis of schizophrenia by the Structural Clinical Interview for DSM-IV (SCID),^22^ 3) psychiatrically stable patients who were about to discharge after remission or who were participating in open wardbased rehabilitation programs, 4) total intelligence quotient (IQ) scores between 80 and 120. Patients with a history of substance use disorders and those with any neurologic and medical disorders known to influence cognitive functioning were excluded. Participants underwent clinical symptom assessments using the Positive and Negative Syndrome Scale,^23^ followed by IQ test, the IGT, and the WCST. Regarding antipsychotic medications, 33 patients were taking stable dosages of atypical antipsychotics (risperidone, olanzapine, or clozapine), nine were taking stable dosages of typical antipsychotics (haloperidol or chloropromazine), and ten were taking therapeutic doses of both atypical and typical antipsychotics.

Healthy participants were recruited from the administrative staff in the two previously mentioned hospitals, and from the community through advertisement. Controls were screened for psychotic, mood, and substance use disorders using the SCID, as well as for history of head injury or neurologic disorder. Among healthy subjects who completed the tests, 55 subjects with IQ's between 80 and 120 were included in the final analysis.

### Intelligence test

An abbreviated form of the Wechsler Adult Intelligence Scale-Revised^24^ was administered that included two subsets, vocabulary and block design. An estimated IQ was calculated by using normative tables.^25^

### Gambling Task

We used a computerized version of the IGT. The same procedures have been described in reports by Bechara.^13^,^26^ In brief, the subjects are instructed that the goal of this game is to win as much money as possible by selecting one card at a time from four decks until 100 cards are chosen; however, the subject does not know when the game will end.

The IGT is a card game that assesses the ability of subjects to evaluate immediate gains over future losses. It involves four decks of cards labeled A, B, C, and D. Selecting card from decks A and B results in a $100 reward, while taking a card from decks C and D results in a $50 reward. However, at sometimes choosing a card from any of the decks may result in punishment. Every set of 10 cards from deck A or B earns $1,000, but costs $1,250 in unpredictable punishment. On the other hand, every set of 10 cards from deck C or D earns $500 but costs $250 in punishment. Therefore selecting cards from decks A and B is disadvantageous because of a net financial loss (-$250/10 cards), while using decks C and D is advantageous due to a net gain ($250/10 cards).

The net difference between reward and punishment in each block of 10 cards was set up in such a way that this discrepancy between reward and punishment in decks A and B was rendered larger in the negative direction across each block. By contrast, this discrepancy between reward and punishment in decks C and D was rendered larger in the positive direction across each block. A net score was then obtained by subtracting the total number of disadvantageous decks from the advantageous decks {(C+D)-(A+B)} for all 100 cards, and for each block of 20 cards.

### Wisconsin Card Sorting Test

The WCST, a commonly used measure of concept formation and flexibility of abstract thought, was administered in a computerized format according to the Heaton protocol.^27^ In this task, subjects sort response cards until they have matched six categories or sorted all 128 cards. Cards are matched on the basis of color, shape, and number, and the rule to which cards are matched changes after 10 consecutive correct card sorts. The sorting principle must be deduced from verbal feedback provided by the computer. Once a particular response mode is established (i.e., 10 consecutive correct responses), a new sorting principle is instituted without warning and must be deduced by the participant. Results typically reported on the WCST include perseverative errors and categories completed. Participant results are felt to most directly reflect dorsolateral prefrontal cortex (DLPFC) function.^28^

### Clinical symptom assessments

Clinical symptom assessments, using the Positive and Negative Syndrome Scale (PANSS), were conducted for patients in the schizophrenia group prior to these tests and on the same day. Symptom severity rating was performed by one experienced psychiatrist.

### Data analysis

The chi-square test (gender) and the t-test (age, education, IQ) were used to compare demographic characteristics between the schizophrenic group and the normal control group. Data analyses of the IGT and the WCST outcome variables were done using t-test and repeated-measures analysis of variance (ANOVA). Correlation between neuropsychological factors and symptoms ratings was tested by Pearson correlation. Data were analyzed with the Statistical Package for the Social Sciences for Windows, Version 12.0 (SPSS Inc, Chicago). All significance was established at 0.05.

## Results

### Subjects

The demographic characteristics are shown in Table 1. There were no significant differences in gender, age, or IQ between groups (p>0.05; all variables). The average amount of education in the schizophrenic group was significantly lower than that in the control group. Symptom assessment at the time of testing revealed low levels of positive, negative, and general psychiatric symptoms in the schizophrenic group.

### Gambling Task performance

Descriptive data for performance on the IGT are presented in Table 2. There was a significant difference in the mean overall net score (advantageous minus disadvantageous deck selection) between groups. The task was then divided into five blocks of 20 card selections to examine changes in performance over time. Chronological card choice in blocks of 20 cards was examined using a 2 (group)×5 (blocks of 20 cards) repeated-measures ANOVA. There were significant main effects for group (F_1,105_=17.73, p<0.001), for block (F_4,102_=24.14, p<0.001), and for the group by block interaction (F_4,102_=8.30, p<0.001).

**FIGURE 1:**
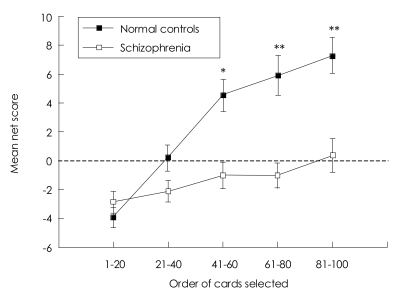
Chronological card choice in five blocks of 20 cards. There were significant main effects for group, for block, and for the group by block interaction. A follow-up independent t-test showed that controls performed significantly better than schizophrenic patients in last three blocks. Data are presented as mean±S.E.M. ^*^p<0.01, ^**^p<0.001.

A follow-up independent t-test showed that controls performed significantly better than schizophrenic patients in last three blocks (block_41-60_; t=3.59, df=105, p=0.001, block_61-80_; t=4.21, df=105, p<0.001, block_81-100_; t=4.04, df=105, p<0.001), but not in first two blocks (block_1-20_; t=-1.04, df=105, p=0.299, block_21-40_; t=1.90, df=105, p=0.060). Even after a correction for multiple comparisons, between-group difference was statistically significant for last three blocks. Healthy participants began by choosing randomly or choosing more from the disadvantages decks in first block and then gradually shifted their choices to the advantageous decks from the second block and reached the plateau at the fourth and fifth blocks. This pattern of shift occurred more gradually in schizophrenia than in normal subjects so that schizophrenic patients could not choose more cards from advantageous decks until the last block (Figure 1).

**FIGURE 2:**
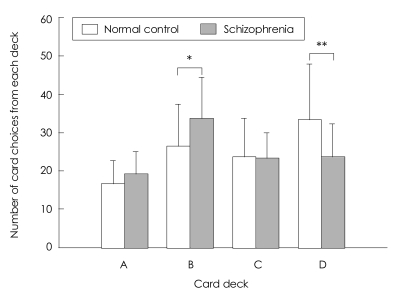
Number of card choices selected from each deck during the 100-card task. Schizophrenic patients selected more frequently from deck B andless frequently from deck D than controls, while there was no difference in selection from decks A and C (after a Bonferroni correction). Regardless of groups, decks B and D were chosen more frequently than decks A and C. Data are presented as mean±SD. ^*^p<0.01, ^**^p<0.001.

The number of choices made from each deck was tested using a 2 (group)×4 (deck) repeated-measures ANOVA. A main effect for deck was found (F_3,103_=25.44, p<0.001), suggesting that subjects as a whole demonstrated a preference for certain decks. Deck B and D were chosen more frequently than decks A and C, regardless of groups. A main effect for group by deck interaction was also found (F_3,103_=11.26, p<0.001). An independent t-test indicated that patients selected more frequently from deck B (t=-3.51, p=0.001), and less frequently from deck D (t=4.16, p<0.001) than controls, while there was no difference in selection from deck A (t=-2.46, p=0.016) and C (t=0.36, p=0.793) after Bonferroni correction (significant if p<0.05/4)(Figure 2).

### Relationship between Iowa Gambling Task and Wisconsin Card Sorting Task

The results of the WCST associated with DLPFC from both groups are presented in Table 2. As is consistent with previous studies, the schizophrenic patients completed significantly fewer categories with more errors than controls. We also tested the correlation between the overall net scores on the IGT and three outcome variables on the WCST within each group and there were no significant correlations for either group (p>0.05, all variables).

### Relationship between Iowa Gambling Task and clinical variables

Pearson correlation indicated that there were no significant correlations between the overall net scores on the IGT and several clinical variables, such as duration of illness as well as positive, negative, and general symptom scores in PANSS (p<0.05; all variables).

## Discussion

The main finding in this study is that chronic stable schizophrenic patients did perform poorly relative to normal controls on the IGT; however, they also showed the slow yet profitable shift from disadvantageous decks to advantageous decks over time. We also found that compared with the healthy controls, schizophrenic patients showed a poorer performance on the WCST, a finding which was not related to the performance on the IGT.

### General performances in response to reward and punishment on the Iowa Gambling Task

The IGT is a test to detect how subject responds to monetary reward and punishment when given in an unpredictable manner. How do schizophrenic subjects react to the reward and punishment? Can he or she get the "hunch" about this task like normal individuals? To the extent of our findings, patients with schizophrenia were less reactive to reward and punishment but did show the shift for card choices from disadvantageous to advantageous decks, in the end. On the contrary, normal subjects began choosing more cards from advantageous decks much earlier, from the second block. These findings are exactly consistent with one previous observation^17^ and broadly consistent with two other previous studies.^15^,^16^

Bechara et al.^29^ reported that the pre-hunch period began about the 10^th^ card, the hunch period about the 50^th^ card, and that the conceptual period at about the 80^th^ card in normal participants. Our normal subjects followed this sequence of card selection, in that their card choices from advantageous decks gradually increased and reached the peak in the last block.

On the other hand, the schizophrenic group did not demonstrate a shift in preference to the more advantageous cards until blocks 81-100. This delayed response to reward and punishment leads to the two assumptions; that schizophrenic patients might be less sensitive to both reward and punishment, or hypersensitive to reward as well as hyposensitive to punishment.^30^,^31^ Ever since Thorndike first presented his view on reward and punishment in 1931, causing obvious and consistent consequences, there have been scores of studies aimed at predicting the performance of schizophrenic patients in the face of reward and punishment. Garmezy^32^ provided a general summary for schizophrenia, showing the atypicality of the response to reward and punishment: 1) the schizophrenic patients' response to punishment tends to become more stereotyped, more inflexible, and less solution-oriented, whereas normal individuals frequently produce more variable behavior, 2) the performance of schizophrenia, following reward, is, at best, equivocal while normal subjects generally produce rather stable effects in maintaining activity. In this regard, impaired sensitivity to both reward and punishment might be more plausible explanation for the poor performance on the IGT; however, the optimal way to test these two possibilities would be to use a variant version of the IGT set in such a way that future reward would increase progressively.^26^

It should be noted that this does not mean patients with schizophrenia are totally insensitive to both reward and punishment, as is the case in those patients with bilateral lesions of the ventromedial prefrontal cortex.^21^

Our findings that schizophrenic patients are less reactive, but still responsive to reward and punishment, have an important clinical implications, in that the token economy for schizophrenia, a treatment intervention based on principles of operant conditioning, have been developed on the basis of this precondition; conversely evident of this treatment's effectiveness has confirmed their partially spared responsiveness to the reinforcers.^33^

### Frequency and magnitude of reward and punishment

From our results about the preference for decks, participants as a whole had a preference for decks with low frequency-high magnitude of punishment (B and D), which means that subjects showed more sensitivity to the frequency than to the magnitude of punishment. This finding is in agreement with two previous reports in both control and patient groups,^14^,^34^ and with one study within only the patient group.^16^ This tendency is corroborated by Greenberg and Weiner^35^ in which the subjects were not influenced by the actual amount that they had won or lost on previous trials, but rather on the ratio of 'wins' to 'losses'. From this and other previous findings, we could speculatively narrow down the difference in performance on the IGT between groups to the matter of the different responsiveness to the magnitude of punishment.

### Physical features of stimuli

The IGT uses artificial money and short sentences such as "You win" or "You lose" with the sounds of slot machine representing reward and punishment. Therefore, the possibility that the natures of those stimuli are enough to provoke schizophrenic patients' responses in a predictable way should be considered. As far as real monetary reward is concerned, schizophrenia showed quite inconsistent behavior to such reward,^36^ suggesting that the artificial money used in the IGT might be not enough to lead them consistent responses. With regard to verbal cues, Buss and Lang^37^ noted that the information from cues had greater importance for psychotic patients in certain tasks, meaning that normal subjects recognize the correctness or wrongness of a response as soon as it occurs whereas schizophrenics seem to be less able to instruct themselves, therefore needing additional informative cues to improve their performances. The sentences used in the IGT, though seemingly evident, are less informative because the subject needs to estimate further that he or she is gaining or losing money considering the net amount of money. Taken together, the experimental task itself such as artificial money and verbal cues might account for the poor performance in the schizophrenic group. However, few studies have systematically investigated whether the feature of a reinforcer has an effect on the performance of this task.^39^

### Relationship with the Wisconsin Card Sorting Test and clinical symptoms

Schizophrenic patients, as compared to healthy subjects, exhibited significantly poorer performance on both the IGT and the WCST. Whereas the result of the performance on the IGT has been inconsistent, the impaired performance on the WCST has been consistently reported in previous studies in chronic schizophrenic patients.^40^ To explain such a difference between the consistencies on the IGT and the WCST and the previous results of intact performance on the IGT in first-episode schizophrenia, Rodriguez-Sanchez et al.^34^ hypothesized that DLPFC functions may be affected by neurodevelopmental processes (i.e. impaired from the onset of the illness) while the orbitofrontal cortex (OFC) could be affected by neurodegenerative processes (i.e. deteriorated with illness progression). Our results seem to partially corroborate their hypothesis, though the result of this study only reflects the cross-sectional status in chronic schizophrenia subjects.

Another consistent previous finding is that performance on the IGT did not correlate with that on the WCST, a finding corroborated by our results.^14^,^16^,^17^,^19^ This supports the hypothesis of Bechara that deficits in decision-making occur independently of deficits in working memory,^38^ making the suggestion of the involvement of working memory in poor decision-making, as mentioned above, appear less plausible.

There was no correlation between clinical variables (PANSS scores of positive, negative, and general) and performance on both the IGT and the WCST. Only one study reported a significant relationship between Scale for the Assessment of Negative Symptoms (SANS) and the IGT total score,^16^ whereas two other studies found no association.^17^,^41^ From this and previous findings, the performance on the IGT seems less related with the symptomatology.

### Limitations

This study has some limitations. Firstly, we did not control for antipsychotic medication in the schizophrenic patients, which could affect their decision-making performance. Beninger et al.^19^ found that 18 patients on atypical antipsychotics demonstrated impairments similar to OFC patients whereas 18 patients on typical antipsychotics did not significantly differ from controls on a decision-making task, stating that the existence of dysfunction on decision-making is related to the kind of antipsychotic medication. Secondly, patients with schizophrenia were not classified into subtypes. While exploratory in nature, one study indicated that the performance of decision-making could be more defective in catatonic schizophrenia than in paranoid schizophrenia.^15^ Thirdly, the study was limited in its interpretation of the nature of impaired decision-making, because we did not use the variant IGT, which was developed to determine whether emotional decision-making deficits may arise from either hyposensitivity to both reward and punishment, so called 'myopia for the future' or hypersensitivity to reward.^30^,^31^

### Conclusion

In summary, we concluded that chronic stable schizophrenic patients with IQs of above 80 did perform poorly relative to normal controls on the IGT; however, more importantly, they also showed the slow yet profitable shift from disadvantageous decks to advantageous decks over time. Based on previous literature, impaired sensitivity to both reward and punishment might be more plausible to the poor performance on the IGT in schizophrenic patients. Specifically, this impairment seems more related to the different responsiveness to the magnitude than to the frequency of punishment, and to the different interpretation of less informative verbal cues in the context of the reinforcing schedule, and less related with working memory and psychological symptoms.

## Figures and Tables

**TABLE 1:**
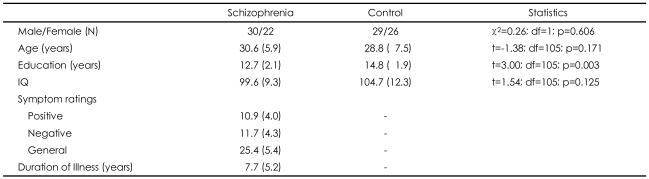
Demographic and clinical information for schizophrenic and control groups

Standard deviations appear in parentheses

**TABLE 2:**
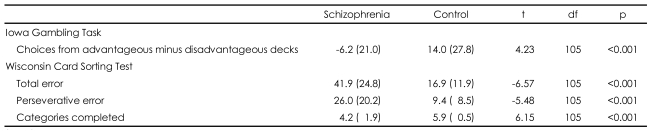
Performance on the Iowa Gambling Task and Wisconsin Card Sorting Test

Standard deviations appear in parentheses
